# Fluorescence microscope (Cyscope^®^) for malaria diagnosis in pregnant women in Medani Hospital, Sudan

**DOI:** 10.1186/1746-1596-6-88

**Published:** 2011-09-24

**Authors:** Saad El-Din H Hassan, Abd Elrahium D Haggaz, Ehab B Mohammed-Elhassan, Elfatih M Malik, Ishag Adam

**Affiliations:** 1 Federal Ministry of Health, P. O. Box 325, Khartoum, Sudan; 2 Faculty of Medicine, University of Geizera, P. O. Box 412, Medani, Sudan; 3 Commission for Biotechnology and Genetic Engineering National Center for Research, P. O. Box 108, Khartoum, Sudan; 4 Ministry of Health, Gezira State, P. O. Box 492, Medani, Sudan; 5 Faculty of Medicine, University of Khartoum, P. O. Box 102, Khartoum, Sudan

## Abstract

**Background:**

Accuracy of diagnosis is the core for malaria control. Although microscopy is the gold standard in malaria diagnosis, its reliability is largely dependent on user skill. We compared performance of Cyscope^® ^fluorescence microscope with the Giemsa stained light microscopy for the diagnosis of malaria among pregnant women at Medani Hospital in Central Sudan. The area is characterized by unstable malaria transmission.

**Methods:**

Socio-demographic characteristics and obstetrics history were gathered using pre-tested questionnaires. Blood samples were collected from febrile pregnant women who were referred as malaria case following initial diagnosis by general microscopist.

**Results:**

During the study period 128 febrile pregnant women presented at the hospital. Among them, *Plasmodium falciparum *malaria was detected in 82 (64.1%) and 80 (62.5%) by the Giemsa-stained light microscopy and the Cyscope^® ^fluorescence microscope, respectively. The sensitivity of the Cyscope^® ^fluorescence microscope was 97.6% (95% CI: 92.2%-99.6%). Out of 46 which were negative by Giemsa-stained light microscopy, 5 were positive by the Cyscope^® ^fluorescence microscope. This is translated in specificity of 89.1% (95% CI: 77.5%-95.9%). The positive and negative predictive value of Cyscope^® ^fluorescence microscope was 94.1% (95% CI: 87.4% -97.8%) and 95.3% (95% CI: 85.4% - 99.2%), respectively.

**Conclusion:**

This study has shown that Cyscope^® ^fluorescence microscope is a reliable diagnostic, sensitive and specific in diagnosing *P. falciparum *malaria among pregnant women in this setting. Further studies are needed to determine effectiveness in diagnosing other *Plasmodium *species and to compare it with other diagnostic tools e.g. rapid diagnostic tests and PCR.

## Introduction

It has been estimated that 90% of the global malaria burden occurs in Sub-Saharan Africa, where during pregnancy 40% women are exposed to malaria infections [1]. Malaria during pregnancy poses a substantial risk to the mother, her fetus and the neonate [2]. Malaria during pregnancy is a major health problem in Sudan, where pregnant women are more susceptible to malaria regardless to their age and parity [3-5] and malaria is associated with poor maternal and perinatal outcomes [5-8].

In the tropics, practitioners are preoccupied by malaria diagnosis, not only in feverish patients but also for many undiagnosed systemic disorders [9]. Such malpractice is not limited to treatment of falsely positive malaria, but presumptive treatment is also frequently practiced [10,11]. Microscopy is the corner stone in malaria diagnosis; it is a valuable technique when performed correctly, but it is unreliable and wasteful when poorly executed. In addition, the technique has its own inherent limitations. The low accuracy of malaria diagnosis is widely recognized in malaria endemic countries [11]. The first step in improving diagnostic tests for parasite-based diagnosis of malaria is of paramount importance. Misdiagnosis of malaria is costly and results in considerable morbidity and mortality, because it contributes to both a delay in treatment of the correct diagnosis and to increasing antimalarial drug pressure and thus resistance, thereby speeding up the obsolescence of affordable drugs [12].

Many diagnostic procedures have been developed to reduce the time, preparation, and training needed to diagnose malaria. The use of *Plasmodium *nucleic acid fluorescent dyes was found to facilitate detection of the parasites even in low parasitaemia conditions due to the contrast with the background [13]. Cyscope^® ^microscope is a mobile, battery-operated microscope with ready slides with malaria parasite DNA specific staining reagents in the dried form. All that is needed is the addition of a drop of blood and viewing the slide under the microscope, saving time and preparation and relatively cheap (£818 for the microscope and £0.40 per test). An optional add-on enables viewing the slides on a computer to facilitate the diagnostic procedure and storage and retrieval of results [14]. However, few published data on Cyscope^® ^microscope in malaria diagnosis are available [13,15-17]. We have recently observed malaria over diagnosis among pregnant women in Medani Hospital [18]. Therefore, this study was conducted to investigate the sensitivity and specificity of the Cyscope^® ^fluorescence microscope in reference Giemsa stained light microscopy as the gold standard in malaria during pregnancy at Medani Hospital Sudan.

## Materials and methods

The study was conducted at Medani Maternity Hospital, Central Sudan during August -October 2010. The area is characterized by unstable malaria transmission and *P. falciparum *is the sole species in the area [19]. Malaria during pregnancy is the common cause of admission to Medani Maternity Hospital, pregnant women are affected by *P. falciparum *malaria regardless to their age or parity and severe case of malaria including cerebral malaria were reported [20,21]. According to the recent surveys, 13 (9.0%) out 146 maternal deaths in Medani Maternity Hospital, were attributed to malaria [8]. Following our recent observations on over diagnosis and treatment of malaria, where there was a poor specificity (8.6%) of malaria microscopy among pregnant women admitted to Medani Hospital we decided to continue investigating the diagnosis of malaria in this setting. The details of the previous study and the specificity of the general microscopist were shown elsewhere [18]. After signing an informed consent, all febrile pregnant women presented to the hospital following initial malaria diagnosis by general microscopist were approached to participate in the study. Socio-demographic characteristics, obstetrics and medical history was gathered using pre-tested questionnaires. A capillary blood (0.5 ml) was taken from each woman into an EDTA tube and was examined using the two methods

### Giemsa stained light microscopy

**Figure 1:**
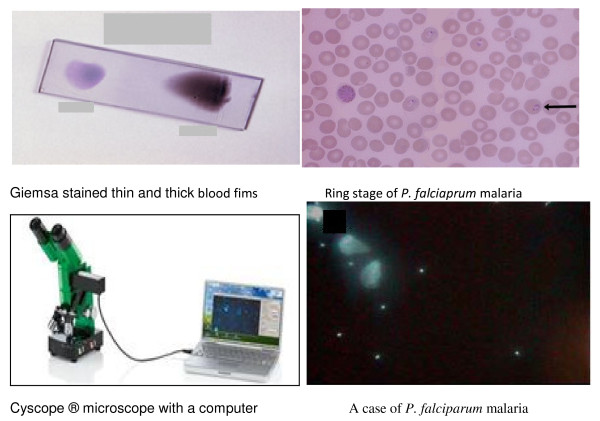
**Blood films/result of Giemsa-stained light microscopy and Cyscope^® ^fluorescence microscope with positive result**.

Thick and thin blood films were prepared and were air-dried and thin film was fixed in methanol, heat fixed and both thin and thick film were immersed in a freshly prepared 10% pre-filtered Giemsa stain solution for 5-10 minutes, washed with water and left to dry, figure 1. Asexual parasites were counted against 200 leucocytes and converted to number of parasites per volume assuming 8,000 leucocytes/μL of blood. Slides were considered negative when no parasites were detected after viewing 100 microscopic fields, figure1. Final microscopy results were based on a rigorous quality control system which included a second microscopist rereading all blood smears and any discrepancies between the first and second readings resolved by a third microscopist. As a quality control cross-check, all slides were transported to Khartoum and re-read at the central laboratory in National malaria control programme to ascertain field accuracy.

### Fluorescence microscope (Cyscope^®^)

The detail of the method was mentioned in our previous work [15] and the test was performed following manufacturer's instructions [14]. In summary it is a microscope that has two light sources: normal light microscopy and fluorescence (UV) light. It uses readily-prepared and ready to-use slides labeled with an unspecific DNA-binding fluorescent dye (4'-6-Diamidino-2-phenylindole (DAPI); emission 443 nm) that detects *Plasmodium *DNA [14]. After mixing the sample and placed on the dye labeled area of a slide with the patients' pathology number, cover slipped, incubated at room temperature for a minute and observed under the 40× objective under UV light (365 nm). The presence of bright shiny tiny dots observed under the UV light indicated the presence of malaria parasites, figure 1. To prevent the slides from drying out, they were kept in a wet chamber. Positive and negative controls were done for each batch of test kits. The person operating the fluorescent microscope was unaware of corresponding light microscopy results.

### Statistics

Diagnostic performance of fluorescent microscopy was established using Giemsa-stained light microscopy as gold-standard. Sensitivity, specificity, positive predictive value and negative predictive value were calculated. Sensitivity of the Cyscope^® ^was calculated as true positives/(true positive + false negatives), specificity as true negatives/(true negatives + false positives), positive predictive value as true positives/(true positives + false positives), negative predictive value as true negatives/(true negatives + false negatives) [22].

### Ethics

The study received ethical clearance from the ethical committee of the Faculty of Medicine, University of Khartoum, Sudan.

## Results

During the study period 128 febrile pregnant women presented at Medani Maternity Hospital. Thirty-three (25.8%) of these women were primigravidae. The admitted characteristics of these women were shown in table 1.

**Table 1:** Basic characteristics of the febrile pregnant women presented at Medani Maternity Hospital, Sudan

Variables	Mean (SD)
Age, years	28.3 (5.5)
Gravidity	3.3 (2.1)
Gestational age, weeks	25.6 (8.5)
Weight, kg	60.4 (5.8)
Haemoglobin, gm/dl	9.5 (1.6)
Parasite count rings/μl	19884.3 (33257)

The Giemsa-stained light microscopy showed that 82 (64.1%) out of 128 pregnant women were positive for *P. falciparum *malaria. The parasite count ranged from 280-23505 with the mean of 19884.3 parasites/μl. There was no case of other *Plasmodium *species. The Cyscope^® ^fluorescence microscope results showed that 80 (62.5%) of these tested 128 samples were positive for malaria. Out of 82 positive tested by the Giemsa-stained light microscopy, 80 have been found to be positive by the Cyscope^® ^fluorescence microscope while two were negative, Table 2. Thus, the sensitivity of the Cyscope^® ^fluorescence microscope was 97.6% (95% CI: 92.2%-99.6%). Out of 46 which were negative by Giemsa-stained light microscopy, 5 were positive by the Cyscope^® ^fluorescence microscope. This is translated in specificity of 89.1% (95% CI: 77.5%-95.9%), table 2.

**Table 2:** Cyscope^® ^fluorescence microscope results compared to Giemsa stained light microscope

		**Cyscope^®^**	**Results**	
**Giemsa stained light microscope results**		**Positive**	**Negative**	**Total**
	
	Positive	80	2	82
	Negative	5	41	46
Total		85	43	128

Of those tested positive by the Cyscope^® ^fluorescence microscope, 80 were positive by the Giemsa-stained light microscopy, while five were negative. This is translated into a positive predictive value of 94.1% (95% CI: 87.4% - 97.8%). Of those tested negative by the Cyscope^® ^fluorescence microscope, 43 were negative according to Giemsa-stained light microscopy while two were positive. This translated into a negative predictive value of 95.3% (95% CI: 85.4% - 99.2%), table 3.

**Table 3:** Diagnostic performance of fluorescent microscopy (Cyscope^®^) using light microscopy as gold-standard

Performance	% (95%, CI)
Sensitivity	97.6 (92.2-99.6).
Specificity	89.1 (77.5%-95.9)
Positive predictive value	94.1 (87.4% - 97.8)
Negative predictive value	95.3 (85.4% - 99.2)

## Discussion

Previously; it has been shown that only 8.6% of febrile pregnant women presented at Medani Hospital after initial general microscopist had *P. falciparum *malaria by referral lab examinations [15]. In the current study light microscopy showed that 82 (64.1%) out of 128 pregnant women were positive for *P. falciparum *malaria. This might be explained by the extensive training to the general microscopists in the area following the previous results and the recommendations.

The main finding of the current study was a high performance in term of sensitivity (97.6%) and specificity 89.1% of Cyscope^® ^fluorescence microscope in comparison with Giemsa-stained light microscopy as the gold standard. A sensitivity of 98.2% and specificity of 98.3% (gold standard: light microscopy) was obtained in cross-sectional facility-based analytical study of the diagnostic performance of the Cyscope^® ^fluorescence microscope in Sudan [15]. Recently, Nkrumah *et al*., reported a high sensitivity (100%) and specificity (97.4%) of the Cyscope^® ^fluorescence microscope among Ghanaian children [17]. However, in neighbouring Uganda in a large sample sized study involving women of child-bearing age and children from rural and peri-urban regions, high sensitivity (92.1% in adults and 86.7% in children) and low specificity (38.8% in adults and 28.6% in children) were observed for Cyscope^® ^fluorescence microscope for malaria examination using Giemsa-stain light microscopy as the gold standard [16]. Thus, application of Cyscope^® ^fluorescence microscope in cross-sectional community-based studies leads to many false positives and this was attributed to small fluorescent bodies of unknown origin mistaken as malaria parasites [16]. However, the false positives in the current study could be explained by the submicroscopic parasitaemia among these pregnant women. It has been shown that over one third of pregnant women in eastern Sudan had submicroscopic *P. falciparum *infections [23]. In the current study there were two false positive cases. Because it was a small sample sized study, stratification of these results by parasite count might not be of value. Previously, the majority of false negatives diagnosed by fluorescent microscopy were of low parasitaemia according to light microscopy (≤400 parasites per μL of blood)[16]. The use of *Plasmodium *nucleic acid fluorescent dyes was found to facilitate detection of the parasites even in low parasitaemia conditions due to the contrast with the background [13]. Recently, the sensitivity and specificity of the Cyscope^® ^fluorescence microscope was 62.2% (95% CI, 56.3 to 67.8) and 96.0% (95% CI, 92.3 to 98.3), respectively using real-time PCR as the gold standard [24].

Generally, the accepted level of sensitivity for a rapid diagnostic test (RDT) in diagnosing malaria is a sensitivity of 95% at a parasite density of 100 parasites/μl [25]. One of the limitations of the current study was that parasite counts were not performed by Cyscope^® ^fluorescence microscope. Recently, it has been shown that parasite count obtained from Cyscope^® ^fluorescence microscope were significantly lower than those obtained from Giemsa-stained light microscopy [17].

Thus, the affordable pricing, portability and compact design of the Cyscope^® ^fluorescence microscope, and the fact that reagents do not require cold storage, make the method a potentially attractive alternative for malaria diagnosis in the rural setting [16]. Furthermore, previous studies confirmed that Cyscope^® ^fluorescence microscope requires very little training and has a short turnaround time of averagely 5 minutes per test [15,16]. Yet, the disability of specific identification and differentiation of *Plasmodia *species as the disadvantage of Cyscope^® ^fluorescence has to be remembered. Thus, Cyscope^® ^fluorescence microscope would be very helpful in areas mainly endemic with *P. falciparum*. Generally RDTs are easy to perform by travelers and even for home management of malaria. Moreover RDTs distinguishes *Plasmodium species *although they are less accurate in this respect than microscopy.

## Conclusions

This study has shown that Cyscope^® ^fluorescence microscope is a reliable diagnostic, sensitive and specific in diagnosing *P. falciparum *malaria among pregnant women in this setting. Further studies are needed to determine effectiveness in diagnosing other *Plasmodium *species and to compare it with other diagnostic tools e.g. rapid diagnostic tests and PCR.

## Competing interests

The authors declare that they have no competing interests.

## Authors' contributions

EBM and ADH carried out the study and participated in the statistical analysis and procedures. SHH, EMM and IA coordinated and participated in the design of the study, statistical analysis and the drafting of the manuscript. All the authors read and approved the final version.
